# Nature-Inspired Enzymatic Cascades: Emerging Strategies for Sustainable Chemistry

**DOI:** 10.3390/molecules31040603

**Published:** 2026-02-09

**Authors:** Eliana Capecchi, Elisabetta Tomaino, Giulia Onnelli, Valentina Ubertini, Raffaele Saladino

**Affiliations:** 1 Department of Ecological and Biological Sciences, University of Tuscia, 01100 Viterbo, Italy; e.tomaino@unitus.it (E.T.); giulia.onnelli@studenti.unitus.it (G.O.); valentina.ubertini@unitus.it (V.U.); 2 Department of Chemistry and Technology of Drugs, Sapienza University of Rome, Piazzale Aldo Moro 5, 00185 Roma, Italy

**Keywords:** enzymatic cascades, artificial metabolism, hybrid chemoenzymatic systems, photo biocatalysis and electrocatalysis, sustainable organic synthesis

## Abstract

Enzymatic cascades, defined here as multi-enzymatic sequences operating on a shared reaction pathway and inspired by the spatial and temporal organization of metabolism, have emerged as powerful and versatile tools for sustainable organic synthesis. They minimize intermediate isolation, enhance atom economy and ensure outstanding chemo-, regio- and stereoselectivity, providing efficient alternatives to conventional multistep routes. Here, we highlight the conceptual role of substrate channeling, minimal cells, artificial metabolism and enzyme promiscuity in the translation of enzymatic cascades into synthetic strategies. Special attention is focused on advanced immobilization on functional and renewable supports, which enhance stability and recyclability and introduce new ways for thermodynamic and kinetic control. Hybrid systems integrating enzymes with photocatalysis, electrochemistry and chemical modules expand the catalytic repertoire far beyond biology. Complementary tools in bioinformatics, structural modeling and artificial intelligence may also enable pathway balancing, predictive design and dynamic optimization. Applications span from the valorization of renewable feedstocks to the synthesis of privileged scaffolds and fine chemicals.

## 1. Introduction

In living organisms, enzymatic cascades are a pervasive solution for complex chemical transformations. It is estimated that hundreds of enzymatic cascades operate in central and secondary metabolism, spanning glycolysis, tricarboxylic acid cycle (Krebs cycle), nucleotide biosynthesis and secondary metabolism. Their unique efficiency arises from the spatial and temporal coordination of enzymes, which prevents the accumulation of intermediates, minimizes energy dissipation and ensures chemo-, regio- and stereoselectivity. These properties make enzymatic cascades a powerful source of inspiration for synthetic organic chemistry, where the challenge is to reproduce complex multi-step transformations in a selective and sustainable way. Comprehensive reviews have previously highlighted different aspects of this topic. They include enzymatic cascade applied to industrial challenges, spatial and structural features associated with metabolons [1], one-pot cascades for natural product synthesis [2,3] and artificial cascades [4,5,6]. Despite these studies, a more integrated perspective unifying biological inspiration with practical synthetic applications is still needed. In this context, the heterogenization of enzymes emerges as a crucial enabling tool for the translation of natural efficiency into sustainable catalysis. The immobilization of an enzymatic cascade on functional polymers, nanoparticles and porous matrices enhances enzyme stability, facilitates the recovery of reaction products and allows the system to operate under one-pot conditions with high atom economy [7,8]. This approach has enabled the green synthesis of drugs and bioactive privileged scaffolds, fundamental to modern drug discovery programs [9]. The present review aims to provide a concept-driven perspective on enzymatic cascade design by bridging the evolutionary logic of metabolism with synthetic organic chemistry. Rather than offering a purely descriptive survey of cascade reactions, we focus on how biological organization principles can be translated into rational and transferable catalytic strategies. Within this framework, the present review differs from prior literature by shifting the focus from the cataloging of cascade reactions to the identification of transferable design principles derived from metabolism. By adopting this perspective, enzymatic cascades are discussed as programmable catalytic systems rather than as isolated synthetic tools. We emphasize how minimal cells, artificial metabolism, enzyme promiscuity and hybrid chemoenzymatic systems may converge to efficient enzymatic cascades as scalable synthetic platforms. Special focus will be devoted to thermodynamics and kinetics, advanced immobilization procedures and the use of computational and AI-assisted design. By tracing the continuum from natural to artificial cascades, we outline how evolutionary principles can be reinterpreted to enable sustainable, selective and industrially relevant transformations.

## 2. Origins of Enzymatic Cascades

Designing cascade reactions with controllable synthetic outcomes, high molecular efficiency and selectivity is one of the great challenges in modern chemistry. Evolution has developed highly efficient solutions to this problem. From prebiotic assemblies to the present day, complex cellular enzymatic cascades have become key tools to manage unstable intermediates, controlling molecular flux and ensuring high selectivity. Minimal cells, first conceived in a prebiotic context, demonstrated that simple vesicles and primitive cofactors could sustain multistep catalysis [10,11]. These insights provide a bridge between abiotic chemistry and metabolism, encompassing the relationships among cofactors, compartmentalization, cell life and replication [12,13]. Similar strategies have been reinterpreted in the laboratory as programmable chemical networks, providing a conceptual and practical way for sustainable transformations [14].

### 2.1. Cell Metabolism as a Source of Inspiration for Organic Synthesis

**Figure 1:**
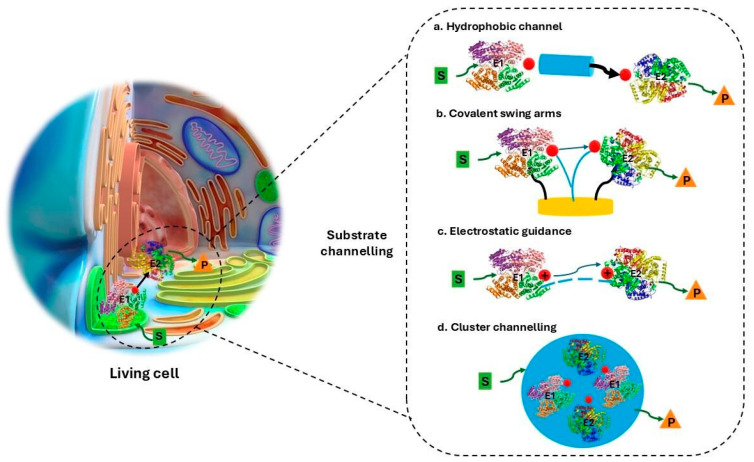
Schematic representation of the main strategies of enzymatic cascades in living cells. The hydrophobic channel (Path a) favors the transfer of intermediates between enzymes. Covalent swing arms (Path b) shuttle intermediates between enzymes in proximity. Electrostatic guidance (Path c) controls the trajectory of electric charged intermediates. Spatial and ordered clusters (Path d) concentrate enzymes in specific microcompartments.

The living cell shows how evolution has turned multistep catalysis into efficient enzymatic cascades. Enzymes rarely freely diffuse in solutions: they are confined within complex assemblies that stabilize intermediates, accelerate molecular flux and prevent undesired side reactions [12]. The key mechanism is substrate channeling, involving the direct transfer of intermediates between the enzymes without the necessity of thermodynamic equilibration in the bulk of the solution [15]. Different evolutionary solutions are recurring in the cell. Quantum mechanical intramolecular tunnelling has been proposed to connect the active sites of enzymes, as in the case of tryptophan synthase, where a 2.5 nm hydrophobic channel transfers indole between adjacent catalytic pockets at a rate of >1000 s^−1^. Electrostatic guidance may steer favorable trajectories for electrically charged intermediates, exemplified by thymidylate synthase-dihydrofolate reductase and malate dehydrogenase-citrate synthase [12]. Covalent swing arms shuttle intermediates in the pyruvate dehydrogenase complex, the lipoamide arm transfers acetyl groups and polyketide synthase employs acyl carrier proteins in a modular assembly-line fashion. Finally, spatial and ordered clusters concentrate enzymes in specific microcompartments (Figure 1). Carboxysomes encapsulate RuBisCO and carbonic anhydrase, mitochondrial super complexes align enzymes in the tricarboxylic acid cycle and flavonoid metabolons assemble at the endoplasmic reticulum [13]. Metabolism also reveals constraints: up to 83% of enzymes are inhibited by metabolites structurally related to natural substrates, and organelle compartmentalization evolved as a solution to mitigate self-inhibition [13]. In addition, enzymatic cascades are not static systems, but they are finely regulated through the action of molecular motors, feedback loops and on/off switching mechanisms. Physical and chemical modulators, including allosteric regulators, post-translational modifications and metabolite-driven signaling, continuously tune the enzyme assemblies, ensuring that molecular fluxes are dynamically adapted to the cellular state [16].

### 2.2. The Minimal Cell

The minimal cell shows that catalytic life-like networks can emerge from simplicity [17]. Pioneered in prebiotic chemistry, minimal cells are described as vesicle-based assemblies where amphiphile molecules, peptides and oligonucleotides act in a combinatorial way to start primitive compartmentalization and informational flow [10]. Recent work has translated these ideas into practical applications. Synthetic minimal cells have been prepared where glucose oxidase and horseradish peroxidase catalyze the polymerization of aniline to polyaniline emeraldine salt (PANI-ES), which spontaneously aggregates into vesicle membranes [18]. Here, the membrane can be viewed as a genotype-like element, encoding the polymer sequence, while PANI-ES feeds back as a phenotype, promoting membrane growth as a synthetic reinterpretation inspired by the central dogma of biology (from nucleic acids to proteins). Primitive cofactors further support this conceptual framework: aromatic heme and Fe–S clusters, plausibly formed under prebiotic conditions as ingredients of the primordial multifunctional organic entity (PriME) [19], confer redox activity when embedded in the membrane, bridging early chemistry with electron-transfer cascades [20]. To overcome protocell fragility, new minimal cells integrate stability and supra molecular communication. Copolymer-stabilized coacervates [21] and multicompartment hydrogels [22] provide selective permeability and robustness, while vesicles equipped with DNA circuits or enzymatic cascades create chemical communication between different receptors, achieving synchronization and the formation of specific reaction patterns [23]. From a practical standpoint, principles derived from minimal cells and metabolon organization have already guided synthetic cascade design through enzyme co-localization, compartmentalization in semi-permeable matrices and diffusion-controlled substrate channeling, enabling efficient and robust one-pot catalytic systems [21]. Thus, the minimal cell is not only a reduced life model but also an envisaged conceptual and technological bridge linking prebiotic chemistry and modern biocatalysis, marking the transition from artificial metabolic networks to feasible natural enzymatic cascades.

### 2.3. Artificial Metabolism

The translation of minimal cell principles into biotechnology enables programmable synthetic frameworks. Artificial metabolism and semi-artificial networks extend these basic principles, encompassing the efficiency of enzyme catalysis, cascade logic and compartmentalization into cell-free and reprogrammed chemical systems. A striking example is provided by an engineered plasmid encapsulated inside *E. coli*, which improves enzymatic clustering and the production of bioactive sesquiterpene amorpha-4,11-diene, a precursor of the drug artemisinin [24]. In this system, sequential enzymes mimic the mevalonate pathway, acting as an intrinsic on/off–like switch to regulate metabolic flux. Early milestones include a seven-enzyme cascade based on fructose-1,6-bisphosphate aldolase for asymmetric C–C bond formation [25] and system-level biocatalytic cycles able to interconvert amino acids and keto acids in modular and functional assemblies [26]. Recent studies show how thermostable [4Fe–4S] enzymes, such as glycerate dehydratase, enable non-natural entry points into cascade chemistry [27]. Improved performance often relies on spatial organization. These strategies encompass enzymes co-immobilized on double-layered hydroxides for sugar phosphorylation [28], protein shells and scaffolds boosting flux in vivo [29] and covalent tagging for enhanced terpenoid production in *E. coli* [30]. In addition, liquid–liquid phase compartmentalization mimics metabolons and improves terpene biosynthesis [31]. Beyond the structural architecture, dynamic control of building block production provides unexpected degrees of freedom in the system: light-responsive protein domains reversibly assemble synthetic organelles in yeast [32], while programmable compartments in bacteria direct phosphoglycosyl transferases with tunable properties [33]. At the microscale, multicompartmental particles produced by gas-shearing may co-localize otherwise incompatible glucose oxidase–catalase cascades reproducing β cell-like behavior in the release of insulin [34]. Artificial metabolism also demonstrates switchable on/off regulation and communication. DNA-based transduction circuits in vesicles enable sender–receiver networks, and vesicle cascades may mimic endocrine-like signaling pathways [35,36]. Biomolecular condensates emerge as programmable platforms where crowding and material properties strongly influence catalysis [37]. Likewise, “mimicomes” combining nanozymes replicate natural complexes with improved stability [38]. The rational assembly of enzymes favored the synthesis of chiral amines, terpenes and alkaloids with high selectivity. In microbial systems, the assembly of enzymes in terpenoid and flavonoid biosynthesis increased the yield of natural substances up to 6.6-fold [39]. Scaffold-free approaches based on short peptide tags such as RIAD/RIDD further enabled modular and tunable assemblies affording carotenoid [40]. The modular assembly of an artificially concise biocatalytic cascade for the manufacture of phenethyl-isoquinoline alkaloids is also reported [41]. These examples clearly illustrate how structural biology, synthetic biology and metabolic engineering may converge to create efficient and flexible synthetic factories. Taken together, artificial metabolism and semi-artificial networks represent an evolutionary continuum: from simple enzyme assemblies to dynamically regulated and communicating scalable systems. By recasting the logic of natural metabolism into modular synthetic frameworks, they provide both a veritable model of life and a versatile platform for sustainable catalysis. Nonetheless, several challenges in scaffold preparation, enzyme stability and long-term functionality remain to be solved in large-scale implementation where catalyst leakage and the costs associated with enzyme purification become critical factors. Future work must therefore focus on optimizing assemblies, balancing confinement and dynamics and integrating hybrid chemoenzymatic strategies to achieve robust and cost-effective platforms for biomanufacturing [42,43].

### 2.4. Benefits and Challenges of Enzymatic Cascades

Enzymatic cascades are attractive because they reproduce key features of metabolism in simplified settings. Their main benefits lie in the direct transfer of intermediates between enzymes, with reduced diffusion in the bulk solution and the stabilization of unstable molecules, avoiding the need for intermediate isolation and tedious purification steps. This principle, often indicated as “channeling”, underpins the efficiency of complexes such as tryptophan synthase and pyruvate dehydrogenase, where tunnels and covalent swing arms ensure controlled molecular flux [44,45]. However, diffusion phenomena dominate in vitro, and the occurrence of spatial proximity alone rarely improves catalysis, highlighting the very fact that efficiency arises mainly from structural integration rather than from a simple enzyme colocalization (Table 1). A second key message from metabolism is compartmentalization. Organelles and metabolons confine reactions, stabilize intermediates and regulate molecular flux through selective permeability. In vitro, enzymes are diluted, and intermediates are prone to degradation, but confinement strategies such as coacervates, vesicles, protocells and phospholipid membranes create selective barriers that improve cascade robustness [18,46]. Cofactor management represents both a benefit and a challenge. Natural pathways rely on the continuous recycling of cofactors (e.g., NAD(P)H, ATP, and SAM), whereas depletion rapidly halts in vitro cascades. Advances in orthogonal regeneration mimic metabolic cycles with notable successes in terpene biosynthesis [47] and the one-pot production of rare sugar nucleotides at an industrial scale [48]. Yet, the stoichiometric balance and long-term stability remain persistent obstacles [45]. A further dimension is switching on/off regulation, which is a central tool for metabolic control. Feedback inhibition, signaling pathways and temporal programs act as molecular switches that finely tune the molecular flux in the cell. In vitro cascades usually lack such adaptive mechanisms, even if recent work demonstrated that enzymatic logic gates can fuel transient pH cycles [49] and that colloidal oxidase assemblies can catalyze their own disassembly [50]. Additional features reveal both potential and challenges in enzymatic cascade. Protein dynamics, spanning from femtoseconds to seconds, govern substrate binding and transition-state stabilization and product release. Their destabilization outside the cell poses a challenge while remaining a design paradigm for next-generation catalysts [51]. Similarly, long-range electron transfer is efficiently managed by natural membrane complexes (STEAP enzymes), which integrate heme and flavin cofactors in very ordered architectures [52]. Reconstituting such molecular order in artificial systems is difficult and critical for sustaining complex redox cascades. Altogether, the benefits and challenges of enzymatic cascades are summarized in Table 1.

## 3. Cascade Design

### 3.1. Assembly and Nanotechnology-Based Supports

**Figure 2:**
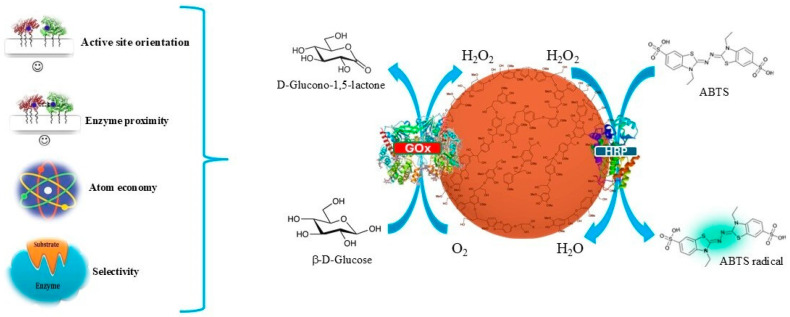
Schematic representation of glucose oxidase–horseradish peroxidase (GOx–HRP) cascade immobilized on lignin nanoparticles (LNPs) [75]. The proximity and correct orientation of the active sites have been ensured by concanavalin A. This configuration assures efficient substrate channeling, atom economy and selectivity. In the first step, GOx catalyzes the oxidation of β-D-glucose to D-glucono-1,5-lactone with the concomitant production of hydrogen peroxide (H_2_O_2_). In the second step, HRP reduces H_2_O_2_ to water, simultaneously oxidizing ABTS into the corresponding radical cation, which provides a detectable colorimetric signal. LNPs preserve enzymatic activity by maintaining optimal distance and orientation, highlighting the role of renewable nanomaterials in the design of efficient enzymatic cascade.

The design of an enzymatic cascade on functional materials represents a critical tool in the translation of catalytical biological efficiency into artificial devices. In metabolism, spatial proximity between the enzymes has evolutionary relevance: enzymes are arranged within metabolons, organelles or multienzyme complexes able to channel intermediates with high local control. Artificial systems aim to reproduce this architectural precision, allowing the exploitation of one-pot sequences and high yields, selectivity and atom economy. Two general strategies have emerged for cascade organization. The scaffold-free strategy exploits the intrinsic ability of enzymes to self-assemble [53]. Cross-linked enzyme crystals (CLECs) and enzyme aggregates (CLEAs) are associated with high stability and activity. Gene fusion, in-frame ligation and engineered interaction domains, such as leucine zippers or SpyTag/SpyCatcher modules, further provide modular ways to co-localize activities without the use of exogenous carriers [54,55]. These assemblies enhance catalytic efficiency by promoting substrate channeling, minimize intermediate escape and constrain the enzymes in specific configurations, with applications spanning from terpenoid and flavonoid biosynthesis to the enantioselective synthesis of amino alcohols via redox-neutral cascades, including systems supported on agarose beads [56]. In these systems, the spatial co-localization of enzymes was shown to enhance the cofactor recycling efficiency and shift reaction equilibria, resulting in up to 80% conversion of alcohol into amines. In parallel, the scaffold-based strategy provides external architectures to organize enzymes with nanoscale precision [57]. DNA and RNA nanostructures [58], including three-dimensional DNA origami [59,60,61,62], allow the programmable spacing of oxidases and peroxidases [63], demonstrating how catalytic efficiency depends not only on proximity but also on scaffold continuity and the topology. For instance, a DNA-based hybrid architecture enables the translocation of molecules as small as 0.8 nm in radius with minimal leakage [64]. Protein-based scaffolds, such as cohesin–dockerin complexes and synthetic cellulosomes, replicate natural clustering for cellulose degradation [65]. Synthetic protein scaffolds based on SpyCatcher–SpyTag chemistry further highlight the role of geometry in the control of kinetic parameters, tuning both substrate affinity and reaction turnover [66,67]. In this latter case, the conversion rates are reported to be 2.4-fold higher than those obtained with the corresponding free bi-enzyme systems, alongside improved pH tolerance and temperature stability [67]. The palette of supports and materials has expanded dramatically. Polysaccharides such as alginates [68], chitosan [69], and agarose [70] have been re-engineered in electrospun fibers and gels characterized by dual-stimuli responsive activity in biomedical applications [71]. Agricultural wastes, biochar and renewable materials like lignin nanoparticles (LNPs) provided green and low-cost platforms with remarkable ability to preserve orientation [72,73,74]. For example, LNPs have been applied for the immobilization of peroxidase and glucose oxidase to design novel biosensors for the determination of glucose [75] (Figure 2). In this system, LNPs enabled stable enzyme immobilization and controlled co-localization, resulting in enhanced operational stability, improved signal reproducibility and sustained catalytic activity over repeated measurement cycles compared to the corresponding free-enzyme system. In addition, magnetic nanoparticles [76,77], nanoporous electrodes [78,79,80] and metal–organic frameworks (MOFs) [81,82,83] have been reported to enable efficient enzyme loading and facile recovery of the catalyst. Particularly striking are polymersome-based catalytic nano-compartments, clustered by DNA linkers into “satellite organelles”, that recreate metabolic communication and improve oxidative cascades [84,85,86,87]. These diverse strategies converge on a central principle: substrate channeling and structural and spatio-temporal regulation [88].

### 3.2. Thermodynamics, Kinetics and Pathway Balancing

**Figure 3:**
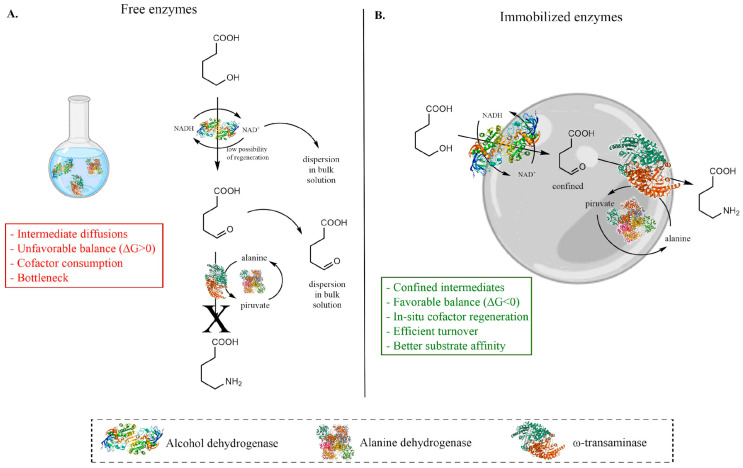
Schematic representation of kinetic and thermodynamic differences between free and immobilized enzyme cascades. Panel (**A**): Free enzymes in bulk solution. Intermediates and cofactors can diffuse and dilute, leading to unfavorable equilibria (ΔG > 0) and potential bottlenecks. Panel (**B**): Immobilized cascade on agarose nanoparticles. Enzymes are confined within a microenvironment that concentrates intermediates and cofactors avoiding their dispersion in bulk, resulting in enhanced kinetics and thermodynamics (ΔG < 0) and faster overall reaction rates [95].

Enzymes anchored on solid supports experience altered enthalpic and entropic contributions and restricted diffusion pathways that affect both ΔG and turnover [89]. These phenomena parallel the logic of heterogeneous catalysis, where adsorption energies and microenvironments dictate activity and selectivity [90] and are equally decisive for the engineering of cascade systems [91,92,93]. In a bulk solution, unfavorable equilibria limit molecular flux, whereas solid supports create microdomains in which intermediates accumulate at high local activities [94]. The co-immobilization of alcohol dehydrogenase, ω-transaminase and alanine dehydrogenase on agarose beads exemplifies this principle via the in situ regeneration of NAD^+^ and the amine donor. The cascade sustains productive molecular flux that would otherwise collapse, highlighting the role of support as an active thermodynamic modulator [95] (Figure 3). Similar effects have been observed with CLEAs, Combi-CLEAs and magnetic derivatives, where confinement of the system reduced the apparent ΔG, enhanced turnover and provided operational stability across multiple classes of enzymes [53,96,97]. Enzymatic cascades also exhibit kinetic behaviors that deviate from the classical Michaelis–Menten law [98,99], especially in the case of immobilized form [100], where substrate affinity is enhanced. A high enzyme packing density, crowding and electrostatic microdomains lead to nonlinear dependencies in which effective molarity becomes the primary driver of the molecular flux [101]. Modeling redox cascades in vivo has revealed unexpected kinetic bottlenecks, showing that enzyme proximity alone does not guarantee optimal throughput [102]. In phosphorylase-based cascades, substrate inhibition emerged as a major limiting factor, but quantitative kinetic analysis allowed the rational extension of substrate loading and improved yields in the case of polysaccharide transformations [103]. These studies underline that the kinetic framework of cascades must be reinterpreted under conditions of confinement and immobilization. The balance of the pathway adds a further level of complexity, since enzymatic cascades require the precise tuning of stoichiometry, enzyme ratios and cofactor economy [104]. In the convergent cascade of cyclohexanone monooxygenase alcohol dehydrogenase, ε-caprolactone is balanced in both the concentration and cofactor turnover [105]. Integrated kinetic and thermodynamic analysis of a multistep cascade for statin side-chain synthesis revealed that pathway imbalance was the primary reason for yield limitations, and fed-batch strategies were proposed to redistribute the molecular flux [106]. Similarly, a combined modelling and experiment-based approach to farnesyl pyrophosphate production identified ATP as the thermodynamic bottleneck, allowing targeted interventions in cofactor supply [107]. Thus, debottlenecking cascades can be viewed as akin to retrosynthetic planning, where equilibrium constraints and kinetic mismatches must be identified and corrected step by step [108]. Beyond case studies, recent innovations propose generalizable design rules. DNA nanostructures demonstrated that catalytic enhancement follows Sabatier-like “volcano” relationships between the scaffold and the substrate [109,110,111]. This data suggests that the interactions between the enzyme and support can be used to tune and to optimize both k_cat_/K_M_ and apparent ΔG.

### 3.3. Enzyme Promiscuity and Noncanonical Reactivities. The Other Side of the Enzymatic Cascade

**Figure 4:**
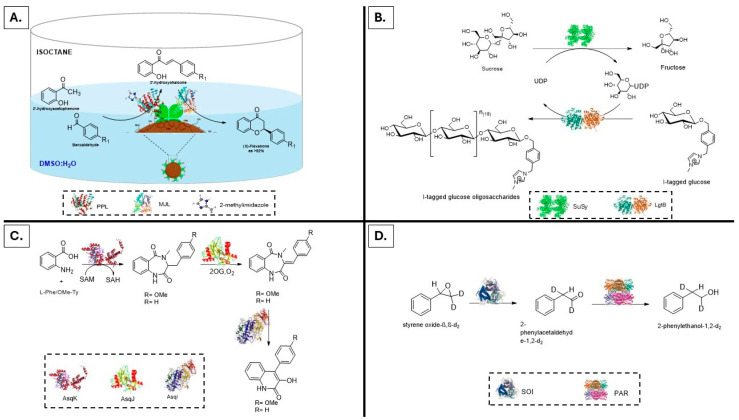
Promiscuous enzymatic cascades and noncanonical reactivities. Panel (**A**): Double-faced lipase cascade affording chiral flavanones: lipase from Porcine pancreas (PPL) catalyzes cross-aldol condensation, and lipase from *Mucor javanicus* (MJL) mediates stereoselective intramolecular oxa-Michael addition. Immobilization on lignin nanoparticles via Concanavalin A enhances activity and reusability [121]. Panel (**B**): Promiscuous galactosyltransferase from *Neisseria meningitidis* (LgtB), in combination with sucrose synthase (SuSy). This system unexpectedly catalyzes glucose polymerization, generating β-1,4 glycosidic linkages [122]. Panel (**C**): Chemoenzymatic platform of Fe (II)/2-oxoglutarate-dependent dioxygenase (AsqJ) and Lewis acid (BF_3_ or AlCl_3_) for the synthesis of viridicatin derivatives [124]. Panel (**D**): Styrene oxide isomerase (SOI) catalyzes the stereospecific Meinwald rearrangement of internal epoxides via 1,2-methyl shifts in cascade with phenylacetaldehyde reductase (PAR) to yield (R)-arylpropanols and amines with high enantiopurity [125].

Enzyme promiscuity refers to the ability of enzymes to catalyze noncanonical reactions and expands the scope of sustainable enzymatic cascades [112,113]. Beyond natural enzyme specificity, promiscuity reveals alternative catalytic landscapes that can be exploited for novel bond-forming strategies [114,115], stereoselective transformations [116,117,118] and hybrid chemoenzymatic routes [119,120]. Unlike a conventional cascade, which emphasizes the optimization of native activities, the exploitation of promiscuity introduces a synthetic flexibility reminiscent of organic chemistry, where reagents are repurposed and reaction manifolds are expanded. In addition, promiscuity can help address challenges related to enzyme availability and cost. A paradigmatic case is that of a lipase-mediated double-faced enzymatic cascade in the synthesis of bioactive chiral flavanones. Two commercially available lipases with distinct promiscuous functions, namely lipase from *Porcine pancreas* (PPL) catalyzing cross-aldol condensation and lipase from *Mucor javanicus* (MJL) promoting stereoselective intramolecular oxa-Michael addition, were combined to afford (S)-flavanones in one-pot conditions and good enantiomeric excess (up to 92%). In this way, the use of poorly available and high-cost aldolases and chalcone isomerase was avoided. Immobilization of this enzymatic cascade on lignin nanoparticles via Concanavalin A further improved the overall performance of the enzymes furnishing a reusable and sustainable heterogeneous biocatalyst [121]. Computational docking and molecular dynamics confirmed the structural basis of the stereochemical outcome, highlighting how promiscuity can be rationalized and guided at the molecular level (Figure 4A). A further example of promiscuity in an enzymatic cascade is represented by glycosyltransferase coupled with sucrose synthase, where galactosyltransferase from *Neisseria meningitidis* (LgtB) revealed unexpected glucose polymerase activity in the formation of β-1,4 glycosidic bonds [122] (Figure 4B). Transformations traditionally confined to organic chemistry have been unlocked through promiscuous activities in enzymatic cascades. The repertoire of promiscuous enzymatic cascades extends into the alkaloid’s synthesis. The Fe (II)/2-oxoglutarate dioxygenase (AsqJ), previously applied for the epoxidation of quinolones [123], enabling cascades that combine enzymatic epoxidation with Lewis-acid promoted rearrangements [124]. This strategy produced gram-scale libraries of viridicatin derivatives (57–91% overall yield) by overcoming the scale-up limitations associated with crude lysates using purified AsqJ, highlighting the practical potential of hybrid enzymatic–chemical cascades for drug discovery (Figure 4C). Finally, styrene oxide isomerase (SOI), which catalyzes the stereospecific Meinwald rearrangement of internal epoxides via 1,2-methyl shifts, coupled with phenylacetaldehyde reductase (PAR) afford (R)-arylpropanols acids and amines with enantiopurity exceeding 97% (Figure 4D).

### 3.4. Chimera Enzymatic Cascades

**Figure 5:**
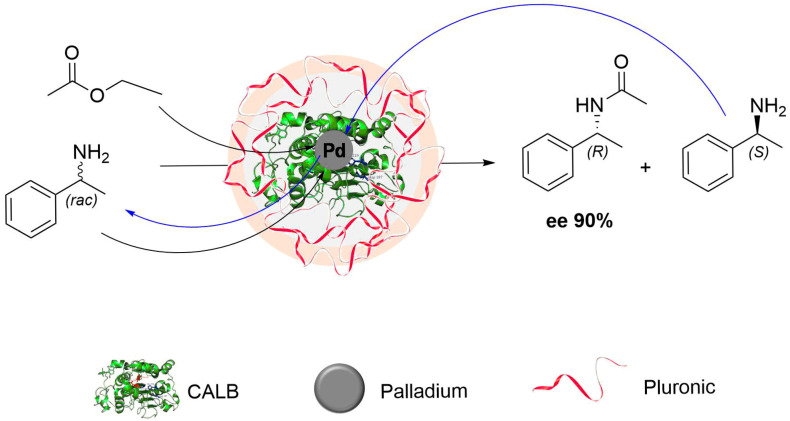
Pd/CALB–Pluronic nanohybrid in the dynamic kinetic resolution (DKR) of racemic 1-phenylethylamine. Pluronic F-127 stabilizes ultrasmall Pd clusters within CALB, enabling concurrent racemization and stereoselective acylation to give enantiopure amides in quantitative yield and high ee [138]. This design also laid the foundation for scalable production through Pd/CALB CLEAs [139].

Chimera enzymatic cascades combine enzymes with inorganic catalysts, metal complexes or artificial cofactors. These systems integrate the stereoselectivity of enzymes with the broad reactivity of transition metals [126,127]. Early studies used free enzymes together with homogeneous metal catalysts in sequential multi-step protocols. They demonstrated compatibility between these components but often showed low robustness and limited synthetic value [128]. Attempts to create more integrated systems included biphasic conditions [129,130] and the application of surfactants or nanomicelles, which reduced catalyst deactivation and enabled one-pot reactions under milder conditions [131,132,133]. A significant advance came with the development of enzyme–metal hybrid catalysts (EMHCs). In these systems, enzymes are immobilized with polymers [134], combined with metal nanoparticles [135], or incorporated into metal–organic frameworks (MOFs) [136]. Immobilization protects enzymes from deactivation by reactive metal species and brings both catalysts into spatial proximity, creating confined environments where cooperative reactivity can occur. One representative example involves lipases co-localized with gold catalysts encapsulated in tetrahedral gallium clusters. This arrangement allowed the synthesis of enantioenriched tetrahydrofurans in high yields, while encapsulation prevented deactivation of the gold catalyst by the enzyme [137]. Simpler and more cost-effective solutions have been realized through Pd/CALB nanohybrids, where lipase B from *Candida antarctica* (CALB) was immobilized on the amphiphilic polymer Pluronic F-127. The polymer creates a nanoreactor that stabilizes ultrasmall Pd clusters [138]. These hybrids perform the dynamic kinetic resolution (DKR) of racemic amines with high efficiency: the Pd clusters catalyze racemization of the undesired enantiomer, while CALB selectively acylates the preferred one. The result is the formation of enantiopure amides in quantitative yield and with enantiomeric excess above 99% under mild conditions (Figure 5). The concept was further developed in Pd/CALB cross-linked enzyme aggregates (CLEAs). In these materials, the enzyme and Pd species are confined within cross-linked aggregates, which improves stability and allows repeated use. The design significantly enhanced productivity, reaching space–time yields reported to be up to five orders of magnitude higher than conventional preparation methods [139].

### 3.5. Analytical, Bioinformatic and AI Tools for Enzymatic Cascades

**Figure 6:**
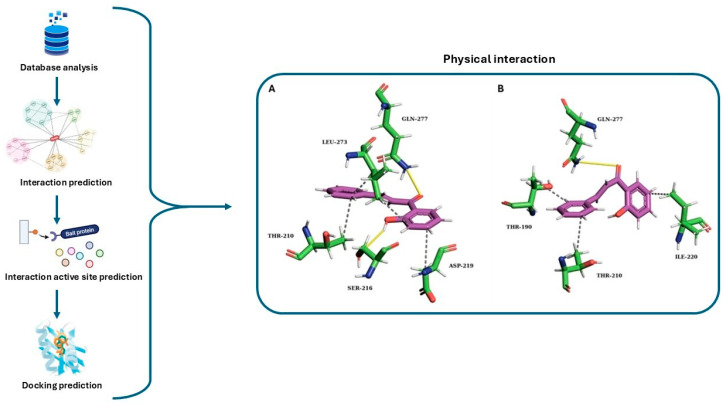
Workflow of computational analysis for enzyme–substrate interactions in the design of optimal enzymatic cascade. The system includes database mining, interaction and active site prediction and docking simulations. Panels (**A**,**B**) show representative docking models highlighting the physical interactions between catalytic residues and the ligand within the lipase active site in the synthesis of stereoselective flavanone [121].

The emergence of advanced analytical, computational and AI-assisted tools is reshaping enzymatic cascade design. These approaches are not auxiliary but represent a core methodology, integrating thermodynamics, kinetics and reaction pathway balancing with predictive accuracy [140,141]. Nanopore sensing, for example, has been applied to resolve, in real time, the concentration–time profiles of substrates, intermediates and products in three-enzyme cascades, distinguishing molecular flux driven by bulk diffusion from confined transfer processes [142,143]. Complementary advances include broadband mid-infrared spectroscopy based on quantum cascade lasers [144] and fluorescence-based probes [145], which extend the classical kinetic toolbox into real-time, high-resolution readouts of enzyme dynamics. At the computational level, bioinformatic pipelines are increasingly used to design balanced and convergent cascade architectures. The recently introduced SubNetX software identifies subnetworks that integrate cofactors, byproducts and thermodynamic constraints within genome-scale metabolic models, generating synthetic routes that mirror the logic of convergent organic synthesis [146]. Sequence-based predictors such as EP-pred accelerate the discovery of broad-scope esterases for chemo- and stereoselective trans-formations [147]. Molecular docking and molecular dynamic simulations have been employed to rationalize the stereoselectivity in enzymatic cascades. A notable case is the double-face promiscuous lipase cascade leading to (S)-flavanones, where docking studies of chalcone intermediates into the active site of lipase revealed the contribution of conserved residues in stabilizing the S-ring-closure pathway. This computational workflow, from database analysis to docking prediction, together with representative enzyme–substrate interactions, is illustrated in Figure 6 [121]. These approaches explain stereoselectivity but also inform about the quality of the immobilization strategy, directly linking silico predictions to experimental implementation. Artificial intelligence further expands these capabilities. Bayesian Optimization (BO) has emerged as a powerful framework for bioprocess design, enabling the simultaneous optimization of solvents, cofactors, enzyme ratios and immobilization parameters in complex cascades [148]. Compared to classical design-of-experiment strategies, BO accelerates discovery, reduces the experimental burden and accommodates multi-objective trade-offs, such as the yield versus E-factor or stereoselectivity versus productivity. From a process-engineering perspective, enzymatic cascades are inherently difficult to optimize because their performance depends on the simultaneous adjustment of multiple interdependent variables, including the enzyme ratios, cofactor regeneration rates, substrate concentrations, pH, temperature and reaction time. In practical cascade operation, mismatched kinetics between individual steps often result in intermediate accumulation, product inhibition and suboptimal space–time yields, ultimately limiting overall throughput [149]. Data-driven optimization strategies provide a practical route to address these constraints by directly operating on experimentally accessible process variables. Bayesian optimization frameworks have been applied to ATP-dependent enzyme cascades to iteratively tune enzyme loadings and cofactor concentrations, achieving higher specific activities and complete product conversion with a limited number of experiments, in some cases outperforming reference systems based on stoichiometric cofactor addition [150]. Such approaches enable the efficient identification of operating windows that balance reaction rates and cofactor economy without requiring detailed mechanistic models.

## 4. Application of Enzymatic Cascades

### 4.1. Redox Cascades

**Figure 7:**
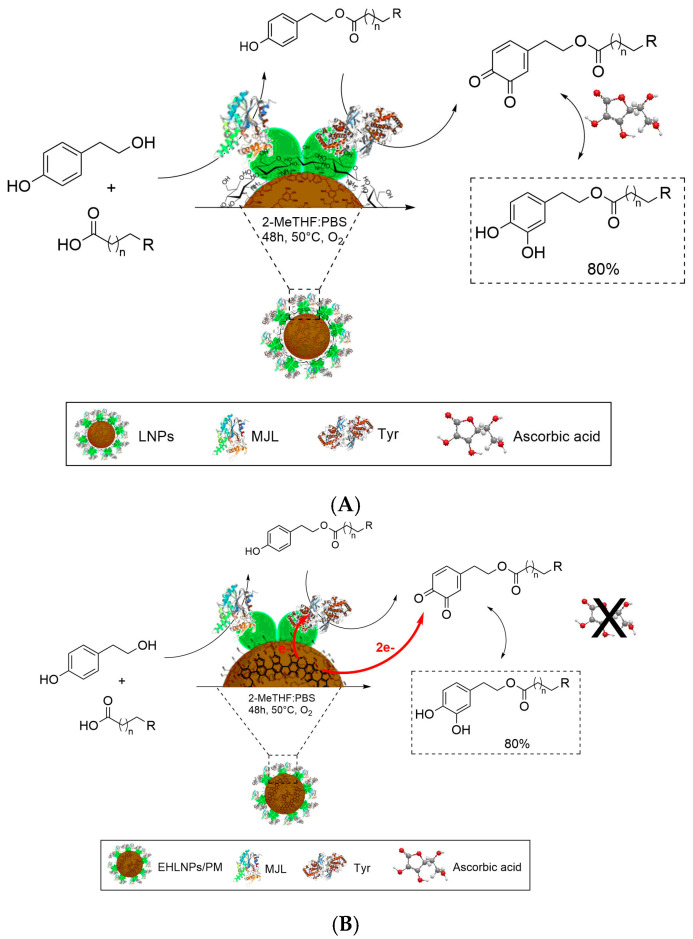
One-pot redox cascades for the synthesis of bioactive hydroxytyrosol esters from simple tyrosol and carboxylic acids. Panel (**A**): Lipase from *Mucor javanicus* (MJL) and tyrosinase from *Agaricus bisporus* (Tyr) supported on lignin nanoparticles (LNPs) afford hydroxytyrosol esters in the presence of ascorbic acid as a reducing agent to prevent quinone side-chain formation [165]. Panel (**B**): The same redox cascade immobilized on electroactive hybrid lignin nanoparticles containing melanin (EHLNPs/PM) in the absence of ascorbic acid [166]. Reaction conditions: 2-MeTHF/PBS, 48 h, 50 °C, O_2_. The system affords hydroxytyrosol esters with up to an 80% yield.

Redox cascades combine oxidative enzymes for the activation of primary oxidants such as hydrogen peroxide, organic peroxides and dioxygen, in some cases associated with the presence of redox cofactors. Recent advances emphasized how redox cascades can transform alcohols, alkenes and ketones into complex chiral scaffolds useful for applications in pharmaceuticals, fragrances and sustainable polymers [151,152]. Advances in this topic encompass the cofactor balance, stabilization of reactive intermediates, stereo-divergence, implementation of modular minimal systems, control of the oxidant supply and generalizable toolboxes and protein engineering in unconventional solvents [153]. A first representative example is the cascade composed of alcohol dehydrogenase from *Lactobacillus kefir* (LK-ADH), enoate reductase from *Pseudomonas putida* (XenB) and cyclohexanone monooxygenase from *Acinetobacter calcoaceticus* (CHMO), which transforms racemic 2-cyclohexenol into ε-caprolactone [154]. This redox cascade shows that the NADPH/NADP^+^ balance emerges as the kinetic bottleneck. Alcohol oxidase from *Colletotrichum graminicola* (CgrAlcOx) coupled to Old Yellow Enzymes transforms geraniol into menthol with high stereoselective control [155,156]. The control in the delivery of primary oxidants is exemplified by alcohol oxidase (AOx) from *Pichia pastoris* coupled with peroxygenase from *Agrocybe aegerita* (rAaeUPO) [157]. In this latter case, AOx oxidizes methanol releasing H_2_O_2_ in situ, which sustains the successive rAaeUPO-catalyzed hydroxylation of ethylbenzene to (R)-1-phenylethanol with enantiomeric excess above 99% and reported turnover numbers above 46,000. A complementary strategy was reported employing formate oxidase from *Aspergillus oryzae* (AoFOx) for the oxidation of methanol, formaldehyde or formate with reported total turnover numbers of 49,000 [158]. The combination between enoate reductases (EREDs) and Baeyer–Villiger monooxygenases (BVMOs) illustrates the topic of stereodivergence and regiodivergence. In this cascade, EREDs reduce (R)- and (S)-carvone to cis- and trans-dihydrocarvone, respectively, which are subsequently oxidized by BVMOs to yield canonical or non-canonical carvolactones [159]. This sequence achieves yields up to 76% with diastereomeric and enantiomeric excess values above 99%. The robustness of the redox cascade can be improved by combining enzyme engineering with specific solvents. Alcohol dehydrogenase (ADH) and cyclohexanone monooxygenase (CHMO) linked by an oligonucleotide linker catalyze the oxidation of cyclohexanol to ε-caprolactone with internal NADPH recycling [160]. In a deep eutectic solvent (DES) composed of betaine and glycerol, the redox cascade displayed higher thermostability, preventing product hydrolysis and maintaining ~97% yields even at high substrate loading. Redox cascades have been also extended to the synthesis of heterocycles. The combination of oxidase (AcCO6) with bifunctional imine reductases (IREDs/RedAms) enables sequential oxidation and the reductive amination step of amine derivatives, affording piperidines, azepanes and 2,5-disubstituted pyrrolidines [161]. The stereochemical outcome was dependent on the IRED variant, opening a one-pot way to a large panel of derivatives with high biological activity. Examples of applications of supported redox cascades in biomedicine are also reported, mainly focused on the localized generation of reactive oxygen species for therapeutic purposes [75,162,163,164]. The immobilization of redox cascades on functional supports can further improve the efficacy of the transformation, especially in the case in which the support furnishes a contribution to the reaction. For example, the one-pot synthesis of bioactive hydroxytyrosol esters from tyrosol and long chain carboxylic acids has been reported using a cascade based on the combination of lipase from *Mucor javanicus* and tyrosinase from *Agaricus bisporous*. To avoid the formation of undesired quinones as side products, ascorbic acid was used as a redox additive [165]. Notably, the use of ascorbic acid was subsequently avoided through the design of redox active support based on lignin nanoparticles containing melanin. Under this experimental condition, the formation of quinones was inhibited by the reductive effect of the melanin component [166]. A schematic comparison between these redox cascades is reported in Figure 7.

### 4.2. Photo Biocatalytic Cascades

**Figure 8:**
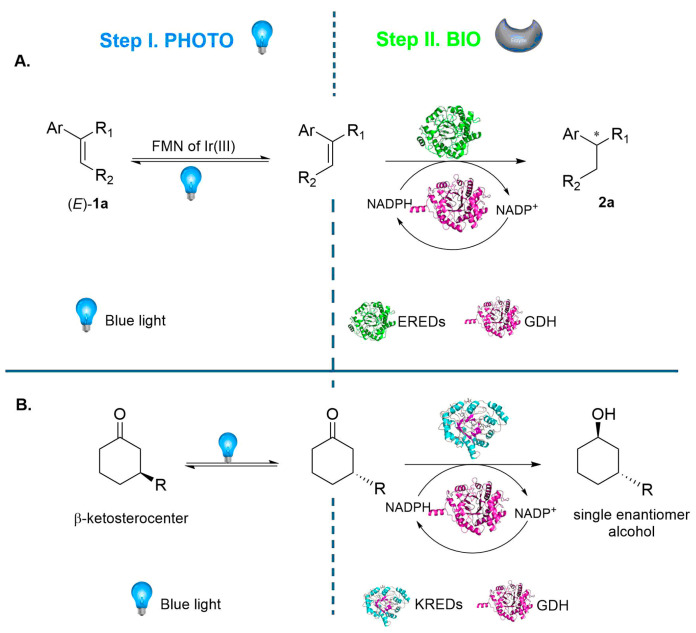
Examples of photo biocatalytic cascades. Panel (**A**): Photocatalytic step: blue-light isomerization of E/Z alkenes (FMN or Ir(III) photocatalyst). Enzymatic cascade: reduction by ene-reductases (EREDs) with NADPH regenerated by glucose dehydrogenase (GDH) [186]. Panel (**B**): Photocatalytic step: blue-light induced racemization of β-substituted ketones. Enzymatic cascade: stereoselective reduction by ketoreductases (KREDs) with GDH-mediated recycling of NADPH, yielding enantioenriched alcohols [187].

Photocatalysis coupled with biocatalysis recently emerged as an advanced frontier in organic synthesis, enabling the design of an enzymatic cascade in which photon-driven processes are connected to multi-enzymatic transformations [167,168,169]. Photocatalysis alone has long been recognized as a versatile tool for C–C and C-N bond formation [170,171,172,173], C–H activation [174,175], oxy-functionalization [176,177] and carbon dioxide reduction [178,179]. The integration of photocatalysis with an enzymatic cascade provided access to novel stereoselective and highly regio specific transformations that would be difficult to achieve otherwise [180,181,182]. Within this framework, photo biocatalytic cascades represent a powerful strategy. A recurring motif in this system is the photocatalytic regeneration of cofactors, such as nicotinamide derivatives (NADH or NADPH), which in turn sustain the oxidoreductase cascade [183,184,185]. One of the first examples of photo biocatalytic cascade is the stereoconvergent reduction of E/Z mixtures of alkenes [186]. Ene-reductase (ERED) is regenerated in the presence of glucose dehydrogenase (GDH) and is able to recycle NADPH. The photo-driven E/Z photoisomerization ensures that both geometrical isomers of the starting materials are transformed into a common intermediate. This combined strategy converts a mixture of stereoisomers of olefins into a single enantiomerically enriched product, thus overcoming the major limitation of the simple biocatalytic step (Figure 8A). A similar approach was applied in the dynamic kinetic resolution of β-substituted ketones [187], in which case, the initial photochemical transformation generates planar radical intermediates, enabling the racemization of otherwise stable stereocenters. In combination with ketoreductases (KREDs) and GDH, this transformation resulted in a fully integrated photoenzymatic dynamic kinetic resolution, where the enzymes reduced one enantiomer and the photocatalytic cycle replenished the racemic pool (Figure 8B). In addition, the photooxidation of alcohols and alkanes to corresponding ketones was realized using sodium anthraquinone sulfonate (SAS), followed by the action of hydroxynitrile lyases (HNLs), benzaldehyde lyases (BALs), aminotransferases (ATAs), ketoreductases (KREDs), ene-reductases (EREDs) and Baeyer–Villiger monooxygenases (CHMOs), in combination with GDH for the regeneration of the cofactor [188]. Rather than defining a single fixed sequence, this approach illustrates a flexible toolkit, where the same photochemical initiation step feeds into multiple biocatalytic cascades depending on the target transformation. In all cases, the resulting one-pot processes deliver enantioenriched products with excellent stereoselectivity and scalability.

**Figure 9:**
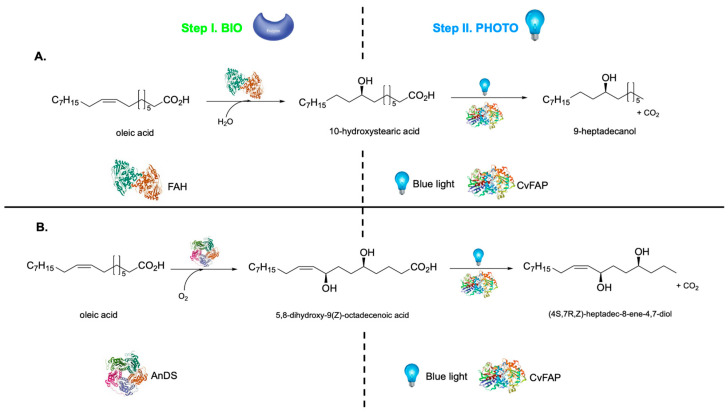
Example of photo biocatalytic cascades based on the first enzymatic step followed by the photocatalytic step [189]. Panel (**A**): Biocatalytic step: oleate hydratase (FAH) catalyzes the stereoselective hydration of oleic acid to 10-hydroxystearic acid. Photocatalytic step: fatty acid photo decarboxylase from *Chlorella variabilis* (CvFAP) performs blue-light driven photodecarboxylation, affording enantioenriched 9-heptadecanol. Panel (**B**): Biocatalytic step: 5,8-diol synthase from *Aspergillus nidulans* (AnDS) hydroxylates oleic acid to 5,8-dihydroxy-9(Z)-octadecenoic acid. Photocatalytic step: CvFAP-mediated blue-light photodecarboxylation yields optically pure (4S,7R, Z)-heptadec-8-ene-4,7-diol.

The biocatalytic and photocatalytic transformations may also be inverted [189]. For example, oleate hydratase (FAH) and fatty acid photodecarboxylase from *Chlorella variabilis* (CvFAP) were combined to achieve stereoselective hydration of the fatty acid double bond, followed by blue-light-driven CvFAP-catalyzed photodecarboxylation of the terminal carboxyl group, yielding enantioenriched secondary alcohols (Figure 9A). As an alternative, 5,8-diol synthase (AnDS) from *Aspergillus nidulans* was applied to hydroxylate the double bond, followed by CvFAP-mediated photodecarboxylation, yielding optically pure diols (Figure 9B). In both sequences, CvFAP provides the crucial light-driven step. The same photo biocatalytic cascade was applied in a process demonstrated at preparative scale for biodiesel production where process intensification through internal illumination enabled dramatic rate enhancement and efficient photon utilization. While this strategy allowed seamless scale-up to industrially relevant reactor volumes, it also revealed enzyme operational stability as a key bottleneck, with CvFAP turnover numbers remaining below 9000 under process conditions, highlighting the need for improved catalyst robustness in long-term operation [190].

**Figure 10:**
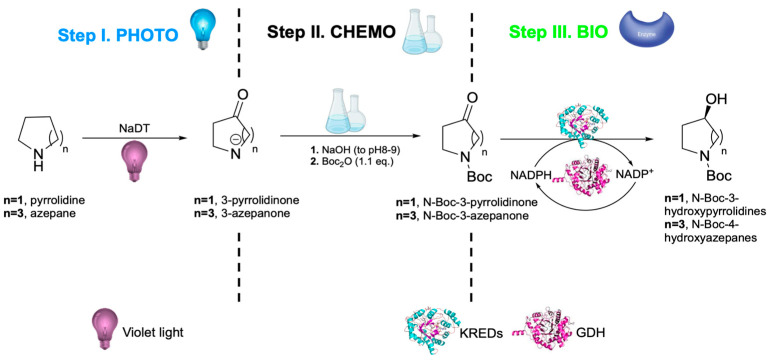
Integration of photocatalysis, chemo catalysis and biocatalysis in a one-pot cascade. Step I (photocatalytic step): violet-light mediated oxidation of cyclic amines (pyrrolidine, azepane) via decatungstate (NaDT) hydrogen atom transfer (HAT). Step II (chemical step): in situ Boc-protection of the corresponding lactams. Step III (biocatalytic step): stereoselective reduction by ketoreductases (KREDs) or aminotransferases (ATAs) with glucose dehydrogenase (GDH) for NAD(P)H recycling, yielding N-Boc-protected hydroxylated amines [191].

Recent work has further expanded the scope of photo biocatalytic cascades through the integration of a chemical step (Figure 10). For example, cyclic amine has been photochemically oxidized by decatungstate-mediated hydrogen atom transfer (HAT) (Step I) followed by in situ chemical Boc protection (Step II) and successive treatment with ketoreductase (KRED) or aminotransferase (ATA) using glucose dehydrogenase (GDH) to supply NAD(P)H for cofactor regeneration (Step III) [191]. Although this system does not constitute a multi-enzyme cascade in the strict sense, since only one enzyme is operative beyond the auxiliary GDH, it exemplifies the maturity of photo biocatalytic one-pot strategies.

### 4.3. Electro Biocatalytic Cascades

**Figure 11:**
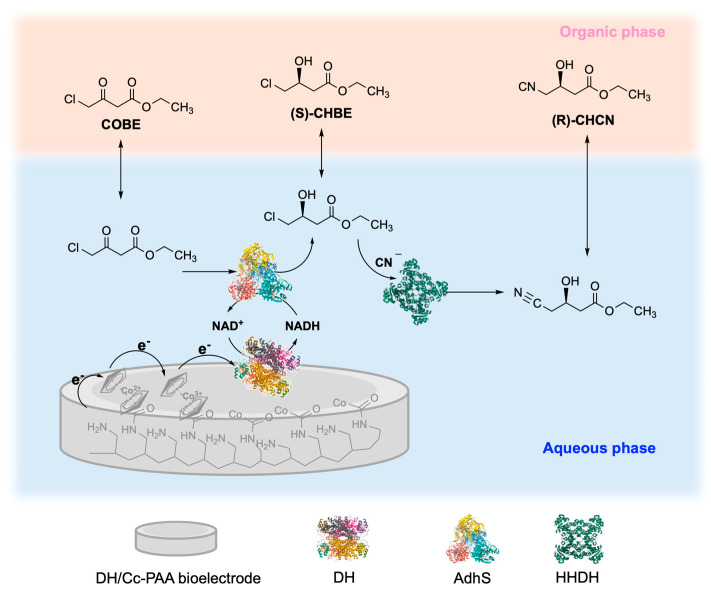
Electro biocatalytic cascade for the asymmetric synthesis of β-hydroxy nitriles. Step I (electrochemical activation): diaphorase from *Geobacillus stearothermophilus* (DH) is immobilized on a carbon–cobalt–polyacrylic acid electrode (DH/Cc-PAA bioelectrode) to regenerate the reduced cofactor NADH from NAD^+^. Step II (enzymatic reduction): (S)-specific alcohol dehydrogenase from *Lactobacillus kefir* (AdhS) catalyzes the stereoselective reduction of ethyl 4-chloroacetoacetate (COBE) to (S)-4-chloro-3-hydroxybutanoate (S)-CHBE. Step III (enzymatic substitution): mutant halohydrin dehalogenase from *Agrobacterium radiobacter* (HHDH) converts (S)-CHBE into (R)-ethyl-4-cyano-3-hydroxybutyrate (R)-CHCN [197].

Electro biocatalytic cascades are systems in which enzymes are combined with electrochemical devices integrating the enzymatic cascade with fine control performed by the electrochemical devices [192,193,194]. Pioneering examples of biocatalysis coupled with electrochemical devices are usually referred to single-enzyme devices [195,196]. Next, electro biocatalytic cascades relied on the combination of two or more enzymes, as for example the case of diaphorase from *Geobacillus stearothermophilus* (DH), (S)-specific alcohol dehydrogenase from *Lactobacillus kefir* (AdhS) and a mutant halohydrin dehalogenase from *Agrobacterium radiobacter* (HHDH) coupled with the electrochemical regeneration of NADH (Figure 11) [197]. This electro biocatalytic cascade was applied in the asymmetric synthesis of β-hydroxy nitriles that are valuable intermediates in the synthesis of bioactive statins. In this framework, DH was immobilized on the electrode surface (bioelectrode), while the two resting enzymes were in the bulk of the solution. In the first step, electrochemical regeneration of NADH at the bioelectrode sustained the AdhS-catalyzed reduction of ethyl 4-chloroacetoacetate (COBE) to (S)-4-chloro-3-hydroxybutanoate (S)-CHBE. Successively, (S)-CHBE was transformed into (R)-ethyl-4-cyano-3-hydroxybutyrate (R)-CHCN by HHDH. A further level of complexity was introduced through the design of a four-enzyme cascade composed by nitrogenase, diaphorase (DI), L-alanine dehydrogenase (AlaDH) and ω-transaminase (HN-ωTA). This cascade was applied in the synthesis of optically pure chiral amines, including bioactive (R)-1-methyl-3-phenylpropylamine [198]. In this latter case, NADH was regenerated electrochemically via the reduction of methyl viologen, which was performed as electron shuttle from the electrode to the enzymatic machinery.

**Figure 12:**
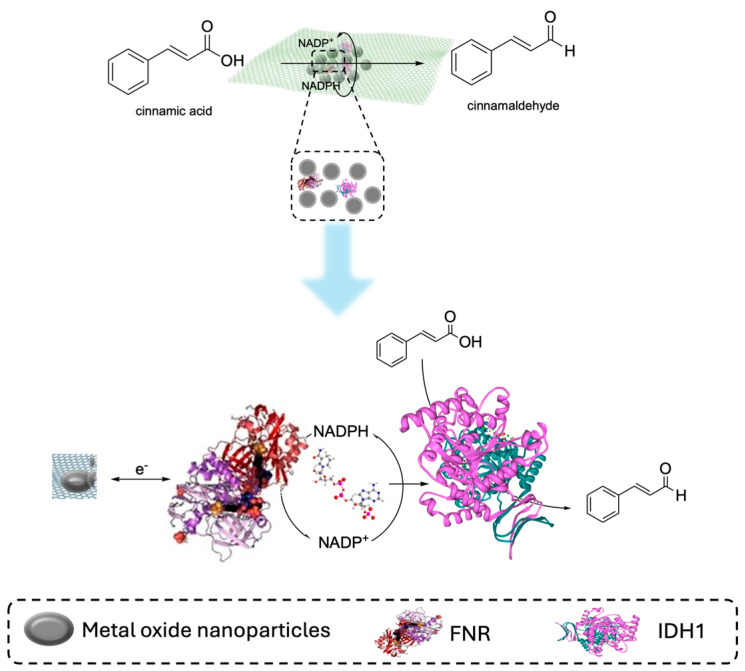
E-Leaf and spatial organization of the enzymatic cascade inside metal oxide nanoparticles [199].

The immobilization strategy has been applied to enhance enzyme stability and reusability in an electro biocatalytic cascade while increasing the local concentration of the cofactor, ultimately leading to higher faradaic efficiency [199]. A representative case of this system is the co-immobilization of ferredoxin–NADP^+^ reductase (FNR) and crotonyl-CoA carboxylase/reductase (Ccr) on a redox-active hydrogel of viologen-modified poly (vinyl alcohol) [200]. In this system, NADPH regeneration is mediated by the redox-active support and facilitates the regio- and stereoselective formation of (2S)-ethylmalonyl-CoA with a faradaic efficiency of 92%. A more advanced strategy included examples of nanoconfinement [201], as in the case of the ordered co-immobilization of ferredoxin–NADP^+^ reductase (FNR) and NADPH-dependent partner enzymes inside mesoporous indium tin oxide (ITO) electrodes. The confined architecture, named electrochemical leaf (e-Leaf), not only sustains continuous NADPH regeneration but also creates a microenvironment that enhances enzyme activity and cascade efficiency (Figure 12). The e-leaf tool was further applied in the interconversion between aspartate and pyruvate and in the reduction of carboxylic acid to the corresponding aldehyde. Electro biocatalytic cascades dedicated to CO_2_ reduction merit special attention due to the implications in environmental challenges [202]. One example is the application of the electro-driven reductive glycine pathway, in which formate dehydrogenase and tetrahydrofolate-dependent enzymes were combined with the electrochemical recycling of NADH, enabling the conversion of CO_2_ and ammonia into glycine [203]. Another environmental-relevant strategy included the application of inorganic catalysts for the initial electroreduction of CO_2_ to formate and CO, followed by enzymatic cascades to transform these intermediates into high-value building blocks for fine organic synthesis [204]. Semi-artificial photosynthesis is produced when electro biocatalysis is coupled with natural photosystems. A prominent example is represented by the Z-scheme architecture in which the photosystem II (PSII) is linked to a tungsten-dependent formate dehydrogenase (FDH) via a dye-sensitized TiO_2_ electrode. In this configuration, the oxidation of water driven by PSII supplies electrons for the reductive conversion of CO_2_ to formate [205].

### 4.4. Synthesis of Privileged Bioactive Scaffolds

**Figure 13:**
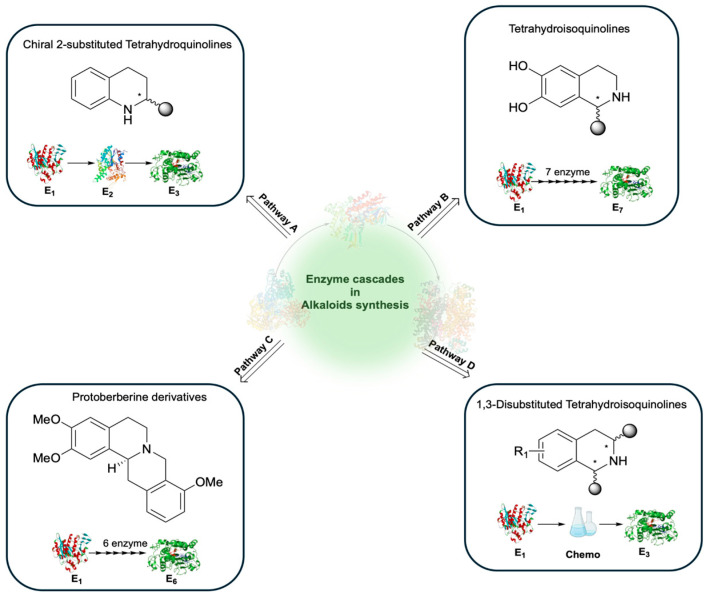
Application of enzymatic cascade in alkaloid synthesis. Pathway A: A three-enzyme cascade enabled the stereoselective synthesis of chiral 2-substituted tetrahydroquinolines [212]. Pathway B: A modular seven-enzyme cascade afforded a panel of methylated norcoclaurine derivatives in high enantiomeric excess [213]. Pathway C: A six-enzyme cascade was engineered to produce protoberberine alkaloids [214]. Pathway D: A modular chemoenzymatic cascade was established for the diastereo- and enantioselective synthesis of 1,3-disubstituted tetrahydroisoquinolines [215].

Remarkable progress in the industrial application of multienzymatic cascades has been reported in the last years. Landmark cases include the route to sitagliptin, atorvastatin and pregabalin [206,207]. In addition, nucleoside analogues such as islatravir, molnupiravir and vidarabine have been prepared via biocatalytic cascades, avoiding extensive protecting-group manipulations and hazardous reagents. These results demonstrate that biocatalysis is not confined to proof-of-concept studies, but it is delivering scalable and sustainable solutions for pharmaceutical manufacturing [208]. Despite these technological advances, access to privileged bioactive scaffolds remains limited. Drug development from natural products is often constrained by the low abundance of these compounds in medicinal plants and by the limited structural complexity and diversity. Attention has therefore been devoted to alkaloid frameworks and benzoxazinoid scaffolds, which are recognized molecular frameworks in library design and drug discovery [209,210,211]. In the framework of alkaloid synthesis, cascade strategies have recently been developed to access structurally complex tetrahydroisoquinoline and quinoline derivatives. A three-enzyme cascade composed of ene-reductase, nitroreductase and imine reductase enabled the stereoselective synthesis of chiral 2-substituted tetrahydroquinolines (Figure 13, pathway A) [212]. A modular seven-enzyme cascade including three distinct methyltransferases afforded a panel of methylated norcoclaurine derivatives in high enantiomeric excess, effectively mimicking the Pictet–Spengler pathway of norcoclaurine synthase (Figure 13, pathway B) [213]. More recently, a six-enzyme cascade integrating carboxylic acid reductase, norcoclaurine synthase, methyltransferases, berberine bridge enzyme and scoulerine 9-O-methyltransferase was engineered to produce protoberberine alkaloids. This system was developed at the gram-scale, following targeted pathway and enzyme engineering to mitigate typical scale-up limitations, and further extended to unnatural halogenated derivatives, underscoring its industrial potential (Figure 13, pathway C) [214]. Additionally, a modular chemoenzymatic cascade was established for the diastereo- and enantioselective synthesis of 1,3-disubstituted tetrahydroisoquinolines, in which the first stereocenter was introduced by a transaminase/acylation step and the second stereocenter by imine reductase, thus enabling divergent access to cis- and trans-isomers (Figure 13, pathway D) [215].

**Figure 14:**
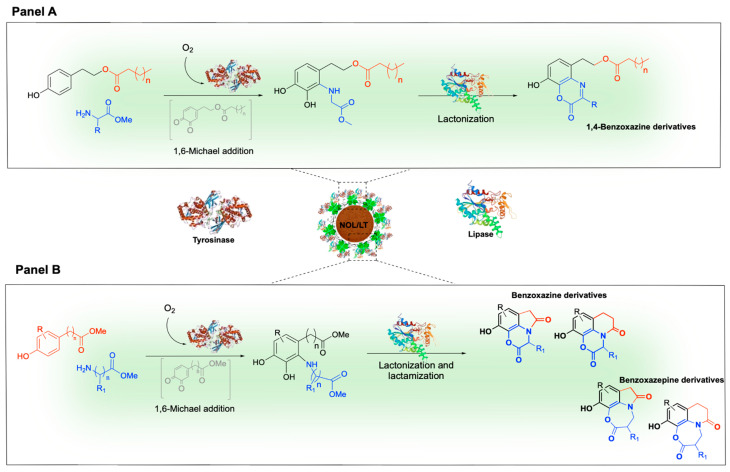
Tyrosinase-lipase cascade for benzoxazines and benzoxazepines one-pot synthesis. Panel (**A**): Lipase/tyrosinase system co-immobilized on lignin nanoparticles (NOL/LTs) performs in a single process the conversion of phenols and α-amino acid methyl esters to 1,4-benzoxazines via sequential ortho-hydroxylation, 1,6-Michael addition, and lipase-catalyzed lactonization [217]. Panel (**B**): Extension of the same cascade to phenolic acid esters to yield tricyclic benzoxazines via lactonization and subsequent lactam formation. Systematic variation in the phenolic side-chain length or substitution of α- by β-amino acid esters direct the cascade toward larger seven-membered lactones, producing benzoxazepine structures [218].

In the synthesis of the benzoxazinoid scaffold, a cascade composed of imine reductases (IREDs) and glucose dehydrogenase (GDH) enabled the stereoselective formation of benzo-fused seven-membered N-heterocycles. 2,4-Disubstituted 1,5-benzodiazepines were obtained in one-pot conditions from 1,2-phenylenediamines and 1,3-diketones, via imine formation followed by IRED-catalyzed reduction. The method afforded excellent yields and stereocontrol (>99% ee and de), demonstrating that free-enzyme cascades provide access to complex benzoxazinoid scaffolds. Comparable frameworks have also been achieved using heterogeneous and recyclable multienzymatic cascades [216]. A coherent retrosynthetic strategy suggests the construction of benzoxazinoid scaffolds from simple phenolic and amino acid precursors. The key disconnection involves an electrophilic catechol-derived quinone, followed by intramolecular annulation to close the lactone and/or lactam rings. This concept has been implemented through a heterogeneous, lipase/tyrosinase system co-immobilized on lignin nanoparticles (NOL/LTs) that operate under one-pot conditions. The cascade sequence involves tyrosinase-catalyzed ortho-hydroxylation, the 1,6-Michael addition of α-amino acid methyl esters and lipase-mediated lactonization, affording 1,4-benzoxazines with high atom economy (Figure 14A). The immobilized biocatalyst is stable and recyclable over multiple runs, ensuring robustness and sustainability. Variation in amino acid esters and carboxylic acids further expands the chemical space of benzoxazines [217]. Extension of the same strategy to phenolic acid esters led to tricyclic benzoxazines, where the presence of two methoxy groups promotes sequential lactonization and the formation of a lactam ring in a telescoped process (Figure 14B). Systematic tuning of the phenolic side-chain length modulates the size and geometry of the additional ring, offering a clear demonstration of scaffold morphing through substituent editing. Switching from α- to β-amino acid esters directs the cascade towards the formation of seven-membered lactones, yielding benzoxazepines. This transformation broadens the structural diversity accessible within the same immobilized platform, thus establishing a unified and sustainable route to both benzoxazines and benzoxazepines, with direct implications for scaffold-based library design and medicinal chemistry [218].

## 5. Conclusions

Enzymatic cascades inspired by metabolism evolved from conceptual models into operational technologies that fully permeate synthetic chemistry. From prebiotic protocells to engineered artificial metabolism, these systems embody the translation of life’s organizational principles—compartmentalization, substrate channeling and dynamic feedback—into controllable and sustainable synthetic tools. Advances in immobilization and the design of functional nanostructured supports, ranging from renewable biopolymers to conductive and photoactive organic–inorganic hybrids, enhance long-term enzyme stability, improve kinetic and thermodynamic profiles and integrate with redox, photo- and electrochemical modules. Together, these achievements define a coherent framework in which enzymatic cascades are no longer considered as isolated bio-transformations, but they become nodes of programmable catalytic networks. The emerging convergence among biocatalysis and material science, artificial intelligence and systems chemistry offers a new paradigm: cascades as adaptive and evolvable systems. Machine-learning algorithms and molecular-level simulations assist in predicting enzyme combinations, balancing cofactor turnover and optimizing spatial arrangements, while micro- and nanoscale confinement brings thermodynamic control comparable to that occurring in living cells. At the same time, hybrid chimera cascades merging enzymes with metal catalysts, photoactive semiconductors and electroactive polymers expand the chemical space far beyond biology, paving the way toward multifunctional platforms capable of performing C–C and C–N bond formations under mild and green conditions. Importantly, the examples discussed throughout this review also highlight the practical constraints that emerge upon scale-up. While several cascades have reached gram-scale production through targeted pathway and enzyme engineering, improved expression and stability or the judicious separation of enzymatic and chemical steps, large-scale implementation remains challenged by catalyst leakage, enzyme deactivation, cofactor economy and the costs associated with protein purification. In this context, data-driven optimization and AI-assisted process control emerge as enabling tools to rationally address kinetic bottlenecks, stoichiometric imbalances and dynamic instability during cascade operation, supporting the transition from laboratory-scale demonstrations to robust and scalable biocatalytic processes. Within this framework, a comparative perspective on external energy inputs becomes increasingly relevant. Photo-driven enzymatic cascades are particularly advantageous when light-induced reactivity and spatiotemporal control enable selective transformations that are difficult to access under purely enzymatic or electrochemical conditions. Electro-driven cascades, in contrast, provide precise control over redox potential and cofactor recycling, often translating into higher energy efficiency, operational robustness and scalability. Rather than representing alternative solutions, photo- and electro-biocatalytic strategies should be regarded as complementary tools whose selection depends on the energetic, selectivity and process requirements of the target cascade. In the coming years, the field will likely move from proof-of-concept demonstrations to effective modular bio-manufacturing, where artificial metabolism is embedded into flow reactors, polymeric matrices and bio-electronic devices for the continuous production of fine chemicals, pharmaceuticals and renewable intermediates. Key challenges remain in enzyme stabilization and viability, cofactor economy and the dynamic control of molecular reaction flux. Addressing these challenges will require the tighter integration of bioinformatics, structural modelling and process engineering, enabling cascades that can self-regulate, adapt and communicate in response to specific environmental inputs. Ultimately, enzymatic cascades represent more than a synthetic methodology; they embody the chemical continuity between life’s origin and modern catalysis. By embracing this continuity, future research can transform cascade catalysis into a unifying platform for green chemistry, capable of connecting biocatalysis, photocatalysis and electrocatalysis within a single, nature-inspired technological vision.

## Figures and Tables

**Table 1:** Natural vs. in vitro enzymatic cascades: features, limitations and message from metabolism.

Features	Natural	In Vitro	Message from Metabolism	Representative Examples
Substrate channeling	Multi-enzyme complexes and tunnels ensure direct transfer of intermediates, preventing build-up of unstable species	Diffusion dominates; spatial proximity alone rarely improves molecular flux	Efficiency comes from structural integration, not mere proximity	Pyruvate dehydrogenase complex; tryptophan synthase tunnel [44,45]
Compartmentalization	Organelles and metabolons confine reactions, stabilize intermediates, and regulate flux via selective permeability	Enzymes diluted, intermediates unstable, prone to degradation	Metabolic flux control requires selective barriers and confined volumes	Protocells, coacervates, vesicles and phospholipid membrane as confinement analogues [18,46]
Cofactor management	Continuous recycling of NAD(P)H, ATP, SAM sustains catalytic turnover	Cofactor depletion rapidly halts cascades	Orthogonal regeneration mimics metabolic cycles	Central carbon metabolism; artificial NAD(P)H regeneration [45]
Switch on/off	Feedback inhibition and signaling pathways act as natural on/off switches	Isolated enzymes lack adaptive feedback; stoichiometry fragile	Metabolic regulation relies on feedback loops and temporal programs	Glycolytic feedback loops; enzymatic logic gates, transient pH oscillations [49,50]
Protein dynamics	Conformational motions tune binding, transition-state stabilization and release	In vitro conditions destabilize dynamics	Design inspired by conformational landscapes	Dynamics of dihydrofolate reductase as paradigm [51]
Electron transfer	Membrane complexes integrate cofactors (heme, FAD) for efficient long-range ET.	Reconstitution outside membranes is difficult and unstable	Precision architecture is essential for sustained redox cascades	NADPH oxidases; STEAP enzymes [52]

## Data Availability

Not applicable.
